# Enhancing men’s engagement in suicide prevention: Integrating masculine norms and personal narratives

**DOI:** 10.1192/j.eurpsy.2025.774

**Published:** 2025-08-26

**Authors:** C.-E. Notredame, T. Delbarre, N. Pauwels, L. Rougegrez, G. Metz, M. Morgiève

**Affiliations:** 1 3114 National coordination center, CHU Lille; 2 Lille Neuroscience & Cognition, Univ. Lille, Lille; 3 3114 National coordination center; 4 Papageno program, F2RSMPsy, St André; 5 3114 Lille regional center, CHU Lille, Lille, France; 6 Department of Health Promotion, Maastricht University, Maastricht, Netherlands; 7 Cermes3, Paris, France

## Abstract

**Introduction:**

In Western countries, men die by suicide three to four times more often than women, a disparity driven by masculine norms that emphasize stoicism and self-reliance. These cultural expectations often discourage men from seeking help, leading them to face their struggles in isolation. Reluctance to seek support significantly reduces access to the social connections that are vital for suicide prevention. Remote services, such as France’s national suicide prevention helpline 3114, offer a discreet and accessible way for men to bypass these barriers and receive the support they need.

**Objectives:**

The national coordination center of the 3114-helpline set out to enhance men’s engagement with its services by developing a strategy that accounts for the impact of masculine norms on their willingness to seek and accept help. To ensure the strategy is relevant and effectively addresses the specific needs of men, it aimed to incorporate insights from those who had been directly affected by suicide.

**Methods:**

A Cyclical Evaluation Process was employed to design and assess this digital public health intervention, comprising three key phases: (1) conducting interviews with men who had contacted the 3114 helpline; (2) developing narratives based on their testimonies; and (3) distributing these narratives through social media and the 3114 website. The stories were specifically crafted to boost readers’ self-efficacy and encourage help-seeking behaviours. This approach triangulates data to evaluate the narratives’ impact on men’s attitudes and behaviours concerning suicide prevention.

**Results:**

Phase 1 involved interviews with five men aged 35 to 51, revealing that hegemonic masculine norms, such as stoicism, significantly impede help-seeking behaviors. In Phase 2, a multidisciplinary team transformed the participants’ testimonies into compelling narratives, which were published between April and May 2024. Phase 3 saw these narratives garner 2,264 website visits and 49,782 social media impressions, indicating strong public engagement. The anonymity provided by telephone helplines facilitated emotional expression, helping to redefine traditional notions of masculinity. The stories played a significant role in raising awareness, normalizing help-seeking among men, and actively dismantling harmful gender stereotypes.

**Conclusions:**

Incorporating the narratives of individuals who have experienced suicidal episodes offers a deeper, more nuanced understanding of this complex issue. When shared through empathetic and respectful approaches, testimonies significantly enhance prevention strategies. Co-constructing knowledge with those directly affected leads to interventions that are more relevant, tailored, and responsive to their actual needs. This approach underscores the ethical imperative of amplifying the voices of vulnerable individuals to develop more equitable and inclusive solutions.

**Disclosure of Interest:**

None Declared

